# *Caenorhabditis elegans*: A Simple Nematode Infection Model for *Penicillium marneffei*

**DOI:** 10.1371/journal.pone.0108764

**Published:** 2014-09-30

**Authors:** Xiaowen Huang, Dedong Li, Liyan Xi, Eleftherios Mylonakis

**Affiliations:** 1 Division of Infectious Diseases, Rhode Island Hospital, Warren Alpert Medical School of Brown University, Providence, Rhode Island, United States of America; 2 Department of Dermatology, Sun Yat-sen Memorial Hospital, Sun Yat-sen University, Guangzhou, China; 3 School of Pharmacy, Second Military Medical University, Shanghai, China; Duke University Medical Center, United States of America

## Abstract

*Penicillium marneffei*, one of the most important thermal dimorphic fungi, is a severe threat to the life of immunocompromised patients. However, the pathogenic mechanisms of *P. marneffei* remain largely unknown. In this work, we developed a model host by using nematode *Caenorhabditis elegans* to investigate the virulence of *P. marneffei*. Using two *P. marneffei* clinical isolate strains 570 and 486, we revealed that in both liquid and solid media, the ingestion of live *P. marneffei* was lethal to *C. elegans* (*P*<0.001). Meanwhile, our results showed that the strain 570, which can produce red pigment, had stronger pathogenicity in *C. elegans* than the strain 486, which can’t produce red pigment (*P*<0.001). Microscopy showed the formation of red pigment and hyphae within *C. elegans* after incubation with *P. marneffei* for 4 h, which are supposed to be two contributors in nematodes killing. In addition, we used *C. elegans* as an *in vivo* model to evaluate different antifungal agents against *P. marneffei*, and found that antifungal agents including amphotericin B, terbinafine, fluconazole, itraconazole and voriconazole successfully prolonged the survival of nematodesinfected by *P. marneffei*. Overall, this alternative model host can provide us an easy tool to study the virulence of *P. marneffei* and screen antifungal agents.

## Introduction

*Penicillium marneffei* is a dimorphic fungal pathogen appearing in mycelia form at 25°C and yeast form at 37°C and is primarily associated with infections in Southeast Asia [1]–[3]. Following the rising prevalence of HIV, penicilliosis has become a significant opportunistic infection in AIDS patients [4]. An experimental murine model was established for the chronic pulmonary and disseminated infection of *P. marneffei* [5], [6], but the ethical and economic factors limited the use of this model. New animal model is urgent to study the pathogenesis of *P. marneffei*.

*Caenorhabditis elegans*, an invertebrate model, is increasingly being used as an infection model to study the pathogenesis of many bacterial and fungal human pathogens. *C. elegans* has emerged as a useful infection model for several reasons, including its easy obtainability, rapid life cycle and physiological simplicity [7]. Also, the nematode shares many morphological similarities with human intestinal epithelial cells and it responds to pathogens in a manner similar to mammals [8]–[10]. The *C. elegans* model has been utilized for several clinically relevant fungal pathogens, including *Candida glabrata*, *Candida albicans*, *Cryptococcus neoformans*, and *Histoplasma capsulatum* [11]–[14]. However, it has not yet been used to study the virulence of *P. marneffei*.

In this study, we developed a *P. marneffei-C. elegans* infection model based on the killing assay, and found that the red pigment and hyphae formation of *P. marneffei* might be crucial toxicity factors involved in *C. elegans* killing. Also, we tested the effect of antifungal agents in this model. This is the first nematode survival assay model in *P. marneffei* infection, and it’s a fast effective screening method for identifying antifungal agents that are active against *P. marneffei*.

## Materials and Methods

### Fungal Strains and Preparation of Conidia

*P. marneffei* strains SUMS0486 and SUMS0570 are clinical isolates got from Sun Yat-sen memorial hospital, Guangzhou, China. The strains were maintained on Potato Dextrose Agar (PDA) plates with 45 µg/ml kanamycin, 100 µg/ml ampicillin, and 100 µg/ml streptomycin at 4°C. To obtain the yeast phase of *P. marneffei*, colonies grown on PDA at 25°C were cultured on brain heart infusion agar (BHI) media at 37°C for 7–10 days. *P. marneffei* conidia, obtained from a culture on BHI plate grown at 37°C for 10–14 days, were collected by flooding the culture surface with PBS and the number of conidia were counted with a hemocytometer.

### *C. elegans* Liquid Killing Assay

*C. elegans* wild-type strain N2 was maintained at 15°C on Nematode Growth Medium (NGM) with *E. coli* HB101 as a standard food source. Stage-synchronized young adult worms were used for all the experiments.30 L4 stage N2 worms were transferred from a lawn of *E. coli* to a 12 wells culture plate containing 2 ml liquid medium of 80% M9 buffer, 20% BHI, 45 µg/ml kanamycin and 10^5^ cells/ml *P. marneffei.* The plates were incubated at 25°C and monitored for their lifespan changes at a 24 h interval. Worms were considered dead and removed away when it showed no response to touch.

### Full Lawn Solid Plate Killing Assay

Swab 200 µl *P. marneffei* culture on BHI plates to perform a fungus full lawn assay. Plates were incubated at 37°C for three days and allowed to equilibrate at room temperature. Approximately 100 adult worms were placed on each plate and incubated at 25°C to monitor their lifespan changes. During exposure of pathogens at different time intervals, observe the phenotypic changes of worms. Test worms were transferred to fresh plates every day to avoid the interference withspawning. Worms were considered dead and removed away from the plates when it shows no response to touch or no pharynx contraction was visible.

### Microscopic Studies

To study the internal colonization of *P. marneffei* in *C. elegans*, nematodes were pre-infected with *P. marneffei* for 4 h. Then the worms were washed three times in M9 buffer and transferred to the fresh medium and incubated at 25°C. At different time intervals, the worms were fixed with 1 mM sodium azide solution and placed on 2% agarose. A confocal laser microscope was used for observation.

### Study of antifungal compounds

To study the efficacy of antifungal agents against *P. marneffei* in this *C. elegans* infection model, amphotericin B, voriconazole, fluconazole, itraconazole and terbinafine were dissolved with dimethyl sulphoxide (DMSO) and added to the liquid assay to the target concentration. The concentration of these antifungal drugs was referenced to the published papers about the antifungal sensitivity to *P. marneffei* [15]. 1.6 µg/ml for amphotericin B, 0.32 µg/ml for voriconazole, 16 µg/ml for fluconazole, 0.8 µg/ml for itraconazole and 3.2 µg/ml for terbinafine. All chemicals were obtained from Sigma (St Louis, MO, USA).

### Statistical analysis

Killing curves were plotted and examined by using the Kaplan–Meier method and differences were determined by using the log-rank test. A *P* value of <0.05 was considered statistically significant. Each experiment was repeated at least three times, and each independent experiment gave similar results. Data presented here are from a representative experiment.

## Results

### 1. Killing C. elegans by P. marneffei

**Figure 1:**
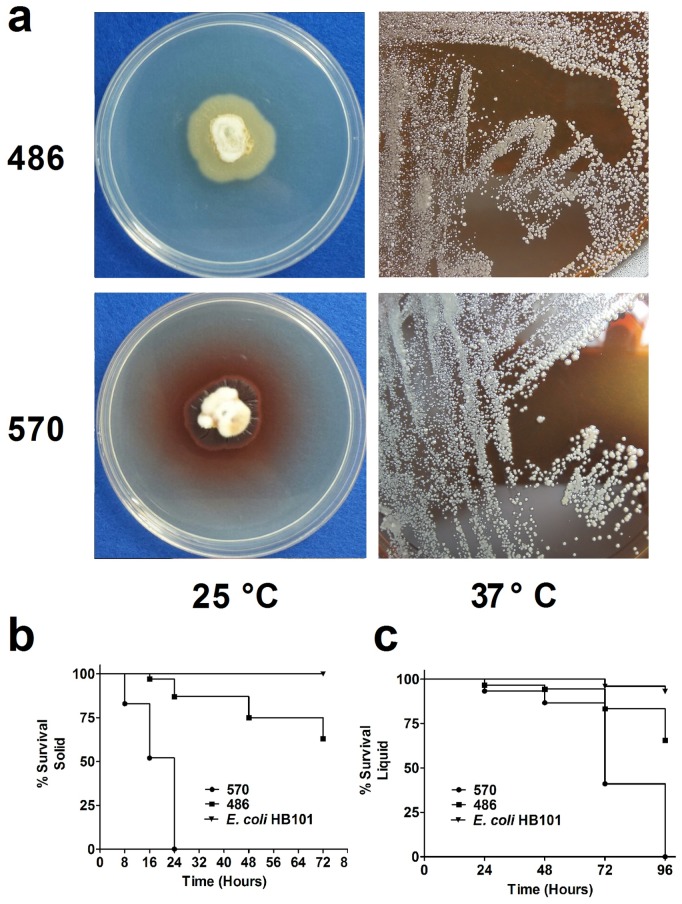
The morphology of two tested *P. marneffei* strains after 7 days of incubation at 25°C and 37°C (a). Survival curves of *C. elegans* after infected with *P. marneffei* in solid medium (b) and liquid medium (c).

Two *P. marneffei* clinical isolates SUMS0570 (570) and SUMS0486 (486) were used in this study. As shown in Fig. 1a, both strains were in mycelial phase at 25°C and yeast phase at 37*°*C. Meanwhile, the results showed that the strain 570 can produce red pigment at 25°C, while the strain 486 does not produce red pigment (Fig. 1a). First, we assessed the virulence of the two strains through the killing of *C. elegans* in both solid and liquid media. For the solid media assay, L4 stage worms were transferred from NGM plates with *E. coli* to BHI plates with *P. marneffei* and monitored for their lifespan changes. The results showed that both strains were able to kill *C. elegans*, with significant difference were found between the strain 486 infection group and *E. coli* control group (*P*<0.001), as well as the strain 570 infection group and *E. coli* control group (*P*<0.001) (Fig. 1b). Interestingly, there was also significant difference between the strain 486 infection group and the strain 570 infection group (*P*<0.001). As shown in Fig. 1b, at 8 h post infection, only worms exposed to the strain 570 began to die, and the mortality rate was 17%. 16 h after infection, it raised to 48%, while there were only 3% worms exposed to the strain 486 died at this time point. In the following 8 h, all worms on the lawn of *P. marneffei* strain 570 died. Similar result was found in the liquid killing assay (Fig. 1c).

### 2. Red Pigment Formation of *P. marneffei* within *C. elegans*

**Figure 2:**
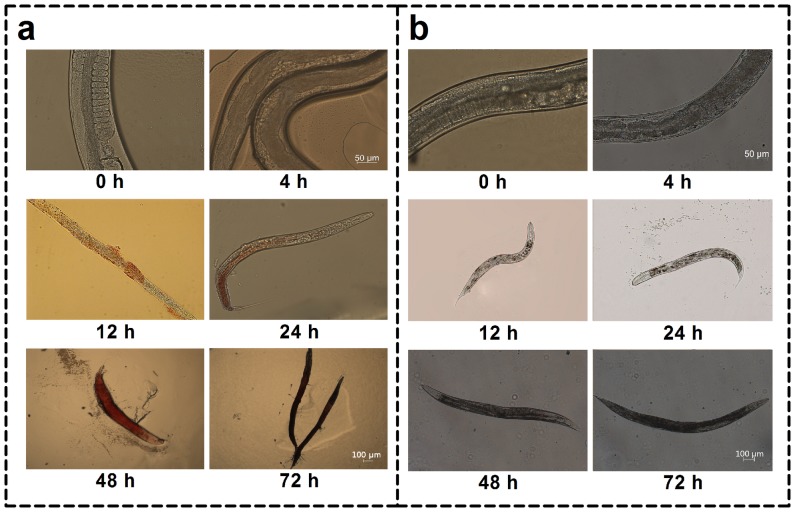
Progression of red pigment in the *C. elegans* intestine. Microscopy of wild-type *C. elegans* N2 at different time points after infected with *P. marneffei* strain 570 or 486. Red pigment was seen in the *C. elegans* swelling intestine and increased gradually with time after infected with live *P. marneffei* strain 570 (a). No obvious swelling or red pigment was seen in the *C. elegans* intestine after infected with live *P. marneffei* strain 486 (b).

The production of red pigment is one of the best-known secondary metabolites produced by *P. marneffei* [16]. Usually, *P. marneffei* exhibits with a characteristic red diffusible pigment at 25°C, and it was reported that the conidia and yeast cells of this fungus could produce melanin or melanin-like compounds *in vitro* and *in vivo* [17]. In this study, we detected the presence of red pigment within *C. elegans* at different time points after infection with both strains. As shown in Fig. 2a, after pre-infection with *P. marneffei* strain 570 for 4 h, the intestine of nematodes extended and during the following 72 h red pigment formed gradually and filled the entire intestine gradually. However, after pre-infection with *P. marneffei* strain 486, no extended intestine or red pigment was found in the nematodes (Fig. 2b).

### 3. Hyphal Formation of *P. marneffei* within *C. elegans*

**Figure 3:**
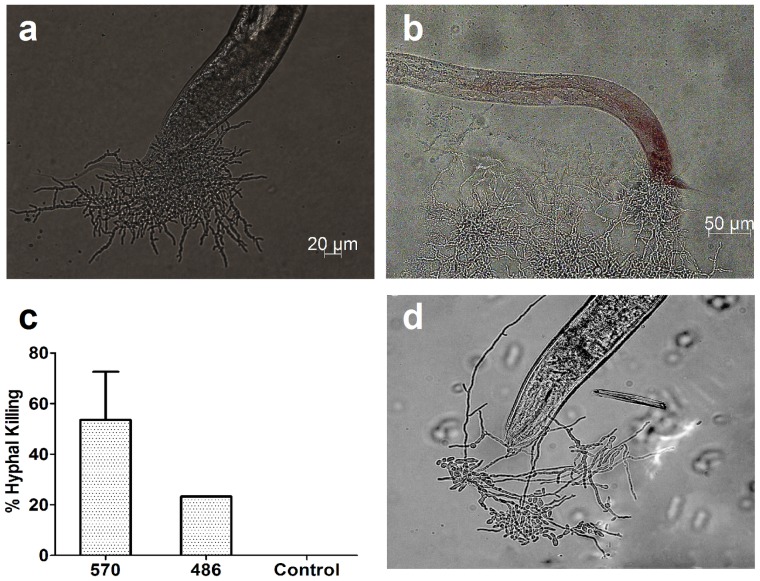
*P.marneffei* hyphae disrupt the nematode cuticle and internal structures. *C. elegans* were pre-incubated with conidia of *P. marneffei* for 4 hours and transferred to pathogen-free medium. Hyphae protruded from the nematode cuticle after infected with *P. marneffei* strain 570 (a, b) or strain 486 (d). Percent hyphal killing of *C. elegans* by different *P. marneffei* strains (c). Images and data were taken 96 hours post infection.

Further, we detected the *C. elegans* intestine injury after *P. marneffei* infection. As shown in Fig. 3a and Fig. 3b, nematodes infected with *P. marneffei* strain 570 demonstrated hyphae had the ability to destroy and penetrate through *C. elegans*. We could see the aggregation growth of hyphae around the broken tail (Fig. 3b). Hyphae were found in about 50% of the infected nematodes (Fig. 3c). When nematodes were infected with *P. marneffei* strain 486, we also observed hyphae in the intestine (Fig. 3d). But there were only 25% worms had hyphal formation (Fig. 3c).

### 4. Utilization of *C. elegans* to study antifungal compounds against *P. marneffei*

**Figure 4:**
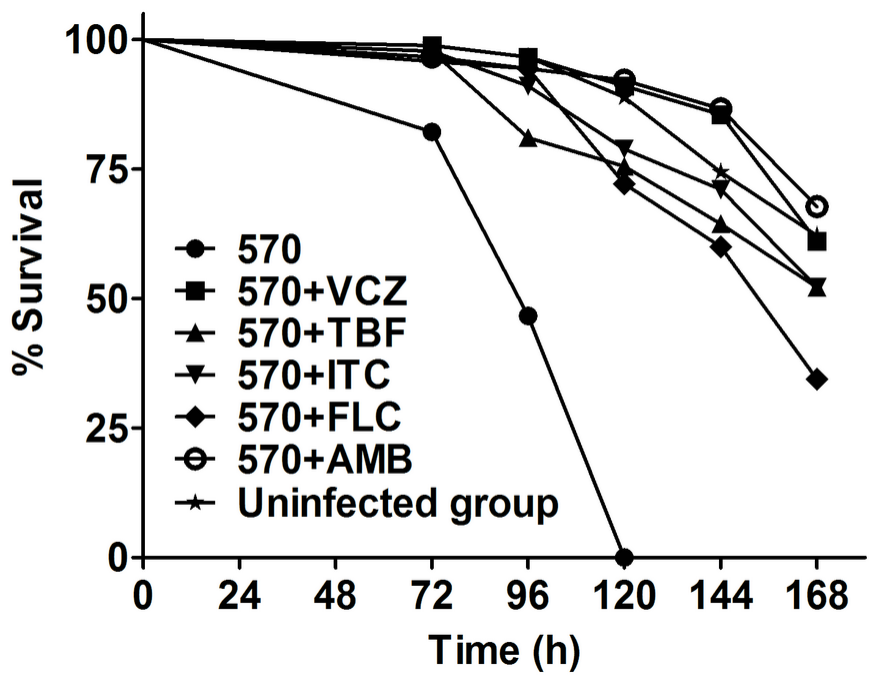
Antifungal agents prolong the survival of wild-type *C. elegans* N2 infected by *P. marneffei* 570. Significant differences were found between 570 infected group and all the administration groups (*P*<0.001).

To assess whether *C. elegans* screen is suitable for evaluating the susceptibility of *P. marneffei* to antifungal agents, 5 antifungal agents including amphotericin B, terbinafine and three azoles (fluconazole, itraconazole and voriconazole) were used in this study. We used the strain 570 as the test strain and found that all 5 antifungal agents prolonged the survival of nematodes infected with *P. marneffei*. The results showed that the mortality of nematodes inthe treatment groupswas significantlylower than the strain 570 infection group (Fig. 4, *P*<0.001 for all antifungal agents compared with the strain 570 infection group). Among different antifungals treatment groups, amphotericin B and voriconazole had the best therapeutic effect while fluconazole had the worst therapeutic effect. As shown in Fig. 4, after administration for 7 days, 1.6 µg/ml amphotericin B or 0.32 µg/ml voriconazole could maintain 60% nematodes survive, while 16 µg/ml fluconazole could only make about 35% worms alive.

## Discussion

The nematode *C. elegans*is a favorable *in vivo* model for a variety of fungal infection disease, and proved to be essential for the study of fungal virulence [7], [18], [19]. In this study, we demonstrated that *C. elegans* was also an appropriate host model in *P. marneffei* infection. Using *P. marneffei* clinical isolate strains 570 and 486, we revealed that in both liquid and solid media, *P. marneffei* conidia ingested by *C. elegans* were able to infect and kill *C. elegans*. Meanwhile, the results showed that the strain 570, which can produce red pigment, had stronger pathogenicity in *C. elegans* than the strain 486, which can not produce red pigment. Further, we found that both strains can form hyphae in *C. elegans*, which might be an important virulence factor of *P. marneffei*. In addition, our results indicated that the *C. elegans*-*P. marneffei* model is reliable to evaluateantifungal agents *in vivo*.

To the best of our knowledge, this is the first report using *C. elegans* model to study the pathogenicity of *P. marneffei*. The *C. elegans* model has been utilized for severalfungal pathogens, including *C. glabrata*, *C. albicans*, *C. neoformans*, and *H. capsulatum* [11]–[14]. However, it has not been used to study the virulence of *P. marneffei*. In this study, using solid method assay, we found the two isolates of *P. marneffei* both had a rapid kill rate, which is significantly greater than that found in *C. neoformans-C. elegans* model [11]. In addition to the solid method assay, we also adopted the liquid method assay. The liquid assay results confirmed the outcome of full lawn assays, but produced less killing during the same time. We supposed that on the solid medium, the fungus had a better growth and nematodes were exposed to more *P. marneffei*. Both these two assays have been used to study host-pathogen interactions, and they have their own characters suitable for different experiments. For example, *C. albicans* forms hyphae in the liquid environment, so it’s better to choose the liquid method assay if the purpose is to study the role of filamentation in virulence [19]. However, in the *C. neoformans-C. elegans* model, solid method assay is mostly applied [11], [20]. Our results indicated that both assays are suitable for studying the virulence of *P. marneffei.*

In this study, we foundred pigment and hyphaemight be two virulence factors of *P. marneffei*. Up to now, the pathogenic mechanism of *P. marneffei* is still not clear. The superoxide dismutase and melanin have been confirmed to be responsible for the virulence in *P. marneffei*. Melanin contributed to the virulence through decreasing the susceptibility of *P. marneffei* to hydrogen peroxide [17]. The yellow pigment of the mold form of *P. marneffei* is composed of mitorubrinol and mitorubrinic acid, which were already confirmed as virulence factors of *P. marneffei* by improving its intracellular survival in macrophages [16]. In this study, the resultsindicated that the formation of red pigment of *P. marneffei in vivo* was associated with its lethality to *C. elegans*, which provided us some clues for further studies of this potential virulence factor. Additionally, it was reported that hyphae was an important virulence factor of *C. albicans* against *C. elegans* [13]. In this study, the hyphae were found in and around the nematode infected with *P. marneffei*, which had the ability to penetrate and destroy the worm’s body. Though the experiment was applied at 25°C, which is different from the temperature of the human body, the results indicated that hyphae formation of *P. marneffei* might contribute to its pathogenicity in host, at least in *C. elegans*.

*C. elegans* was reported to be a powerful tool to screen the antifungal activity [20]. Julia Bregeret al. [13] screened a chemical library using this model for compounds that can prolong the survival time of worms under *C.albicans* infection, and their results were consistent with the results in murine model of candidiasis. However, there has not been report about the antifungal drug susceptibilities of *P. marneffei* in this model. Besides, the referenced *in vitro* method of CLSI documents M27 and M38 have not been used in studies of thedimorphic fungi. In this study, the effect of antifungal agents against *P. marneffei* was easily reflected through the survival time of nematodes. All the five drugs significantly prolonged the infected nematodes’ life. According to the results, we conclude that the *C. elegans*-*P. marneffei* model is suitable to evaluate antifungal agents.

Collectively, we demonstrated that *C. elegans* was an appropriate host model to study the pathogenicityof *P. marneffei* and to evaluate antifungal agents. It can beinferred that *C. elegans* is an easy and desirable model to study the virulence mechanisms of *P. marneffei* and screen antifungal agents.
